# Thermal stability, storage and release of proteins with tailored fit in silica

**DOI:** 10.1038/srep46568

**Published:** 2017-04-24

**Authors:** Yun-Chu Chen, Tristan Smith, Robert H. Hicks, Aswin Doekhie, Francoise Koumanov, Stephen A. Wells, Karen J. Edler, Jean van den Elsen, Geoffrey D. Holman, Kevin J. Marchbank, Asel Sartbaeva

**Affiliations:** 1 Department of Chemistry, Claverton Down, Bath, BA2 7AY, UK; 2 Centre for Sustainable Chemical Technologies, Department of Biology and Biochemistry, Claverton Down, Bath, BA2 7AY, UK; 3 Department of Biology and Biochemistry, Claverton Down, Bath, BA2 7AY, UK; 4 Department of Chemical Engineering, University of Bath, Bath, BA2 7AY, UK; 5 The Medical School, Framlington Place, Newcastle upon Tyne, NE2 4HH, UK

## Abstract

Biological substances based on proteins, including vaccines, antibodies, and enzymes, typically degrade at room temperature over time due to denaturation, as proteins unfold with loss of secondary and tertiary structure. Their storage and distribution therefore relies on a “cold chain” of continuous refrigeration; this is costly and not always effective, as any break in the chain leads to rapid loss of effectiveness and potency. Efforts have been made to make vaccines thermally stable using treatments including freeze-drying (lyophilisation), biomineralisation, and encapsulation in sugar glass and organic polymers. Here for the first time we show that proteins can be enclosed in a deposited silica “cage”, rendering them stable against denaturing thermal treatment and long-term ambient-temperature storage, and subsequently released into solution with their structure and function intact. This “ensilication” method produces a storable solid protein-loaded material without the need for desiccation or freeze-drying. Ensilication offers the prospect of a solution to the “cold chain” problem for biological materials, in particular for vaccines.

Denaturation is a loss of structure and function in proteins through disruption of the network of noncovalent interactions, which maintain secondary, tertiary and quaternary structure. This affects the storage of proteins in solution and is particularly significant for medications such as vaccines, which must generally be stored and distributed through a continuous network of refrigeration at 2 to 8 °C, called the “cold chain”^1^,^2^,^3^. Loss and inactivation of vaccines through breaks in the cold chain are a serious issue for global public health, in particular for mass childhood vaccination programmes in the developing world^2^,^4^,^5^. Considerable efforts have been made to produce more thermally stable vaccines and proteins through approaches including freeze-drying, sugar glass, nanopatch, biomineralisation^6^,^7^,^8^,^9^, pegylation and polymer-microsphere encapsulation^10^,^11^,^12^.

Organisms such as nettles, diatoms and radiolaria make use of nanoscale silica structures for protection^13^,^14^,^15^. They control the deposition of silica by secreting organic molecules, such as the silicateins – positively charged lysine-rich polypeptides – produced by marine sponges. Preformed silica nanoparticles have been suggested as vehicles for drug delivery^16^, and porous silica/protein monoliths have been produced for use in analytic or catalytic columns. Recently developed “imprinting” approaches^17^, using both silica and polymers to define protein sites with shape recognition, have shown that silica can be deposited around proteins and closely match their shape. A recent study of conformational change in haemoglobin made use of a silica matrix to trap structures in different conformational states^18^, and encapsulation in mesoporous silica has been shown to enhance protein stability against heat and denaturation^19^,^20^,^21^. We have therefore explored the storage of proteins in a silica network – covalently deposited by sol-gel methods to entirely surround a protein and render it thermally stable by physically preventing denaturation and unfolding – and their subsequent release back into solution. Our results show that ensilicated proteins not only survive conditions of heat and aging which would denature the unprotected protein in solution, but also can be released with their structure and function intact. As test subjects we have used hen egg white lysozyme (HEWL), a robust and well-characterised protein with enzymatic activity; horse haemoglobin, a heterotetrameric protein with a complex tertiary and quaternary structure; and tetanus toxin C-fragment (TTCF)^22^, a vaccinogenic tetanus fragment, which is a part of the commonly used DTP vaccine.

The ensilication and release process is shown schematically in Fig. 1 and described in detail in Methods. A solution of silica precursor materials (pre-hydrolysed tetraethylorthosilicate (TEOS)) is added to the protein solution, and stirred for 20 minutes. Sol-gel precipitates are rapidly formed, as shown in Fig. 2a, and then vacuum filtered. Precipitates retained on the filter are washed with Milli-Q water and methanol in order to remove any free protein adhering to the surface. Collected ensilicated powders are left to dry in an extractor for 24 hours, and then weighed. We have subjected ensilicated proteins to treatments including heating to 100 °C under dry and wet conditions, and aging for up to six months at room temperature. Silica is specifically vulnerable to attack by acidic fluoride solutions^23^. We therefore use a release protocol involving treatment with a dilute solution of sodium fluoride, acidified to pH 4.0 using HCl, to release the ensilicated proteins into solution. We assess protein concentrations in solution using the standard BCA protein assay. We assess the retention of function (enzymatic activity) in lysozyme using EnzCheck assay, normalising to the protein concentration to obtain specific activity, while for TTCF we make use of ELISA binding assay. These techniques, as well as an array of structural investigations including circular dichroism (CD), Dynamic Light Scattering (DLS), atomic force microscopy (AFM), Fourier Transform Infrared Spectroscopy (FT-IR), Thermo-gravimetric analysis (TGA) and SDS-polyacrylamide gel electrophoresis (SDS-PAGE) are all described in Methods.

Ensilication of 100 mg of lysozyme produces on average 182.68 ± 7.18 mg of powder. Assessment using BCA protein assay indicates that 93.0% ± 2.3% of the lysozyme initially present is successfully ensilicated (Supplementary Table S1). The ensilicated material forms and precipitates rapidly. We were, however, able to obtain some Dynamic Light Scattering (DLS) data on particle size during the process, as shown in Fig. 2b. An initial solution of lysozyme shows peaks corresponding to particles 4 nm in diameter, which correspond to individual lysozyme molecules. After the addition of silica, the 4 nm signal declines and a signal corresponding to larger particles with diameters around 200 nm appears within tens of seconds, showing that silica precipitation on the protein occurs immediately after the addition. This 200 nm peak is not observed in TEOS before hydrolysation or in TEOS gelated without the addition of protein (Supplementary Figure S1). Further analysis of the ensilicated material by atomic force microscopy (AFM), confirmed the presence of globular silica nanoparticles of 200 nm in size (Fig. 2c). Fourier Transform Infrared Spectroscopy (FT-IR) spectra for native lysozyme, silica and the ensilicated material are presented in Fig. 2d. We observe both amide peaks from lysozyme^24^ and silica vibrational bands^25^, showing that the protein and silica exist together in the precipitated material.

Results on the retention of structure and function in lysozyme, after release using our acidic fluoride protocol, are shown in Fig. 3. Enzymatic activity is well retained in our released protein (between 75 to 100% of the activity of the native lysozyme – Fig. 3a), even when the ensilicated material was treated with acid (10 M HCl), stored for six months at room temperature (22 °C), or heated at 100 °C for 5 h. Native lysozyme heated to 100 °C in aqueous solution, by contrast, is denatured and loses function as expected. Acid treatment with 10 M HCl likewise destroys the activity of lysozyme in solution (Supplementary Table S2). The ensilicated material itself shows very low enzymatic activity before the protein is released (Supplementary Figure S2) whereas a control sample of lysozyme deposited on porous silica gel (see Methods) shows full activity. This confirms that in the ensilicated material the lysozyme is encapsulated within the silica, inaccessible for its substrate and protected from chemical or physical attack.

Mass spectrometry analysis on lysozyme before and after ensilication detected a single peak at 14305 Daltons (Supplementary Figure S3), indicating that the protein chain remains intact during ensilication and release. Circular dichroism (CD) analysis confirmed that the ensilicated lysozyme displays the same CD signal as the starting materials, while the unprotected protein subjected to heat treatment shows dramatic changes in the CD signal, indicating loss of secondary structure (Fig. 3b). SDS-polyacrylamide gel electrophoresis (Fig. 3c) shows that native lysozyme heated to 100 °C in solution loses structural integrity (Fig. 3c, lane 7), with the presence of both lower molecular weight protein fragments and higher molecular weight complexes; a lysozyme dimer band is visible at approx. 27 kDa. The ensilicated and released protein, by contrast, appears identical to the starting material. We were also able to crystallise lysozyme released after ensilication and obtain its three-dimensional structure using X-ray diffraction (see Methods and Supplementary Tables S1 and S2 and Supplementary Figure S4). Structural alignment showed that this structure is almost indistinguishable from published structures of native lysozyme (Fig. 3d) with an overall backbone root-mean-square deviation of 0.09 Å.

Since the ensilicated material is produced by precipitation from solution, we expect water molecules to still be associated with the protein. The drying process for our powder is not severe and is not intended to desiccate the sample entirely. Thermogravimetric analysis (TGA) confirms that an ensilicated lysozyme sample loses 8.9% ± 0.1% of its weight in the temperature range 50–100 °C (Supplementary Figure S5), giving us an estimate of the water content.

Having established that ensilication, preservation and release are possible for lysozyme, we have also applied our protocol to horse haemoglobin (Hb) and tetanus toxin C-fragment (TTCF). Protein assay on the supernatant after ensilication confirmed that some Hb and TTCF remained in solution and that up to 46% of the Hb and 72% of the TTCF was ensilicated. The FT-IR analysis of the ensilicated Hb confirmed the co-presence of silica and protein (Fig. 4a). The lower ensilication efficiency for Hb and TTCF compared to lysozyme may be due to the differences in size and charge of the proteins. The interaction between proteins and silica is primarily noncovalent, with polar interactions between silanol groups and charged or polar amino acid side chains being very significant^26^, and Hb differs substantially from HEWL in both its size (Hb 64 kDa, HEWL 14.3 kDa) and its isoelectric point (Hb 6.5, HEWL 9.4). This suggests that the ensilication protocol will in general have to be adjusted for different materials, e.g. by varying concentration and pH. However, ensilication does appear effective in preserving the ensilicated material from heat denaturation. CD analysis confirmed that the Hb’s secondary structure is preserved through ensilication, heat treatment and release, whereas heat-treated Hb without protection displays an obvious and dramatic loss of structure (Fig. 4b).

TTCF is a particularly interesting case in that it is a currently used vaccine component. To test the integrity of the TTCF protein we assessed its antibody binding capacity using ELISA binding assays (Fig. 4c) and observed that heating the ensilicated powder at 80 °C for 2 h and then releasing TTCF did not damage the protein. In contrast, the unprotected protein was completely denatured and lost its antibody binding capacity. SDS-PAGE analysis showed that the ensilication and release procedure did not change the molecular weight of TTCF (Fig. 4d), whereas TTCF heated to 80 °C in solution shows evidence of protein aggregation. We note that TTCF can be lyophilised, rendering it more stable, and have made use of this as a comparison to the ensilication process. Lyophilised TTCF reconstituted after ambient storage or after dry heating (at 80 °C for 2 h) displays an antibody binding capacity slightly below that of native TTCF or ensilicated and released TTCF (Supplementary Figure S6). We may therefore say that ensilication provides a level of protection for TTCF at least equal to lyophilisation, without the need to remove water.

The acidic fluoride release protocol used in this study was intended to establish that the release of intact protein was possible. Treatment using either fluoride or acid separately did not dissolve the silica or release proteins. Experiments monitoring the release of ensilicated lysozyme at different pH values (pH 6.0 to 2.0) demonstrated that a pH of 5.0 or lower in combination with sodium fluoride was necessary for release of the proteins (Supplementary Table S3), while the specific activity of the released protein decreased with decreasing pH; pH 4.0 provided the optimal combination of released quantity and activity. Since fluoride in solution is toxic at high concentrations, causing gastrointestinal distress at a dose of around 100–150 mg^27^, biological applications may require investigation of alternative release methods or the removal of fluoride from solution, for example by adding a soluble calcium salt to precipitate insoluble CaF_2_. We are currently investigating alternative methods including physical disruption of the silica matrix.

Our results demonstrate for the first time that proteins in solution can be encased in a covalently bonded silica network and subsequently released back into solution, intact and functional. The ensilicated protein survives heat treatment that denatures proteins in solution, indicating that the silica is effective in physically preventing protein unfolding and denaturation. The process produces a solid (particulate) protein-loaded product directly from solution, and may thus be suitable for use with proteins that do not tolerate lyophilisation; the product has substantial intrinsic water content. The ability to keep proteins intact in solid form until they are needed would be valuable for the storage of industrial enzymes, vaccines and biological therapeutics, such as antibodies and antivenom treatments. The ensilication process we describe here has the potential to transform vaccine availability worldwide by elimination of the cold chain.

## Methods

### Sources of materials

TEOS, hen egg white lysozyme, horse haemoglobin were all from Sigma-Aldrich.

### Expression and purification of His-tagged TTCF

The N-terminal histidine tagged tetanus toxin *C* fragment construct in pET16B vector was a kind gift from Dr Kevin Marchbank. The fusion protein was expressed in BL21(DE3) *E. coli* strain and purified according to the method described by Hewitt *et al*.^28^ except that the purification was performed on an AKTA FPLC system (GE Healthcare) using a HisTrap HP column (GE Healthcare). Purified protein was extensively dialysed against 50 mM Tris-HCl pH 7.

### Ensilication protocol

500:500:1 of Milli-Q water, TEOS and 32% HCl were stirred for 1 hour at 20 °C for TEOS pre-hydrolysation. Protein solution was prepared as a 100 ml mixture of 1 mg/ml protein in 50 mM Tris-HCl pH 7, stirred using a 25 × 8 mm octagonal magnetic stir bar (Fisher Scientific) at 60 rpm in a 250 ml beaker for 0.5 h at 20 °C. The pre-hydrolysed TEOS was then added to the protein solution with a ratio of 1:50, and stirred at 125 rpm for 20 min. After 20 min, the mixture was vacuum filtered using a Microfibre Filter MF 300 with 0.7 μm retention (Fisher Scientific). Once supernatants filtered from the protein ensilication were collected, gels were washed with MilliQ water and methanol thoroughly in order to remove any non-ensilicated protein left on the surface. Collected ensilicated protein powders were left to dry in an extractor for 24 h, and then weighed.

A control experiment using lysozyme absorbed onto silica was also performed. For this 100 mg silica (SIGMA-Aldrich, Davisil, Grade 646, pore size 150 Å, 35–60 mesh) was soaked with 100 ml 1 mg/ml lysozyme in 50 mM Tris-HCl pH 7.0, for 20 min at 20 °C. The treated silica was then vacuum filtered and left to dry overnight in the fume hood. The supernatant from the filtration was collected.

### Treatment protocols – heat, acid and aging

To test whether the ensilication protects against intense heat, ensilicated powders were heated at 100 °C between 2 and 5 h in the cases of lysozyme and Hb (whose T_m_ values exceed 70 °C). TTCF is rapidly inactivated above 60 °C; for TTCF we therefore used a treatment temperature of 80 °C. The pure lysozyme and Hb proteins were solubilized in 50 mM Tris-HCl pH 7.0 at a final concentration of 1 mg/ml and heated at 100 °C for the same length of time. To test the resistance to acid the powdered ensilicated lysozyme was incubated for 3 h in 10 M HCl. The powdered ensilicated lysozyme samples were stored at 22 °C for 6 months to test stability against aging.

### Release protocol

To release protein from silica, 5 ml of 50 mM Tris-HCl pH 7.0 and 5 ml release buffer (190 mM NaF in Milli-Q water and adjusted to pH 4.0 with HCl) are mixed with 5 mg of ensilicated protein powder in a tube rotator at 20 °C for 1 h.

### Protein concentration assay

The protein concentration was measured using a BCA protein assay kit (Thermo Fisher) according to the manufacturer’s instructions.

### Dynamic Light Scattering (DLS)N

A Malvern Zetasizer Nano ZS was used to measure the hydrodynamic size by dynamic light scattering (DLS) for both lysozyme and to follow the process of lysozyme ensilication. The lysozyme sample had a count rate of 228.8 kcps, using a measurement position of 3 mm, attenuator 11 and duration 60 seconds at 25 °C. To monitor the onset of the ensilication process, we used DLS to measure particle size before (0 sec) and immediately after adding 4 μl pre-hydrolysed TEOS to 200 μl 1 mg/ml lysozyme solution at every 20, 30 and 40 seconds.

### Lysozyme activity assay

We used an EnzChek^®^ Lysozyme Assay Kit from Life Technology following the manufacturer’s instructions.

### Crystallisation, X-ray diffraction data collection and 3D structure determination of released lysozyme

Crystallisation of lysozyme released from silica was achieved with use of the hanging drop vapour diffusion technique^29^. Released lysozyme at a concentration of 25 mg/ml in 0.1 M sodium acetate pH 4.6^30^ was crystallised in 1.5 M NaCl in 0.1 M sodium acetate pH 4.6^31^. Crystals suitable for X-ray diffraction analysis formed after approximately 5 days incubation at 18 °C.

Crystals were flash frozen in a loop (reservoir solution + 25% glycerol) under a continuous nitrogen cryo stream (Oxford Cryosystems Cobra) and full data set was collected on an in-house rotating anode X-ray source (Rigaku MicroMax-007HF) with a Saturn 944 + CCD detector (Supplementary Table S1 for data collection statistics). The structure of released lysozyme was resolved using molecular replacement (using Balbes) and refined (using Phenix) with model building in COOT (Supplementary Table S2 for refinement statistics).

### Atomic Force Microscopy (AFM)

Atomic force microscopy was performed at the European Synchrotron Radiation Facility (ESRF) at Partnership for Soft Condensed Matter (PSCM), Grenoble, France. Samples were prepared by adding 400 μl prehydrolysed TEOS into 20 ml of 1 mg/ml lysozyme solution. After 30 seconds, 10 μl of the mixture was transferred onto a mica. The mixture on the mica was quenched with 0.05 M Tris-HCl pH 2.5 to stop the ensilication process and left for a further 5 minutes before being rinsed with 0.05 M Tris-HCl pH 7 to remove any un-bound material. Images were acquired with an Asylum Cypher AFM, with a scan size of 30 μm in X&Y and with a Numerical Aperture of 0.45.

### Fourier Transform Infrared Spectroscopy (FT-IR)

FT-IR spectra between wavelength 4000 cm^−1^ and 600 cm^−1^ were accumulated from 25 scans with a resolution of 2 cm^−1^, data interval of 0.5 cm^−1^ and a scan speed at 0.2 cm/s on a Perkin Elmer Frontier FTIR spectroscope.

### Circular dichroism

Synchrotron radiation circular dichroism spectra for lysozyme were collected at the Diamond Light Source, Didcot, on beamline B23 over a wavelength range of 180 to 260 nm with an integration time of 2 s and a data interval of 1 nm. For hemoglobin, a Chirascan™ CD Spectrometer (Applied Photophysics) was used with the same parameters as at the synchrotron. Proteins were dialysed into 100 mM sodium phosphate buffer pH 7.0 and protein concentration was adjusted to 0.1 mg/mL. The samples were run in 0.5 mm quartz cuvettes at 20 °C.

### SDS-Polyacrylamide gel electrophoresis (SDS-PAGE)

Protein samples were prepared in SDS-sample buffer and loaded on a 10% or a 15% tris-glycine SDS-polyacrylamide gel. Protein bands were visualised with Coomassie Blue stain.

### ELISA binding assay

Native TTCF, or TTCF after ensilication and release, was coated at 10 μg/ml onto 96-well flat-bottom plates (High-binding plate, Greiner) in ELISA binding buffer (50 mM NaHCO3, pH 9.6) and incubated overnight at 4 °C. The plates were washed four times with PBS and blocked with 1% casein in PBS supplemented with 0.05% Tween 20 (PBS-T) for 1 h. A two time serial dilution of anti-TTCF (clone 10G5) mouse monoclonal antibody^32^ (starting concentration 1 μg/ml) was added and incubated for 1 h at room temperature. Plates were washed further four times with PBS, followed by incubation with HRP-conjugated rabbit anti-mouse IgG (1:10,000 dilution, Thermo Scientific) for 1 h at room temperature. The plates were developed using TMB substrate and the reaction was stopped with 1 M H_2_SO_4_. Absorbance at 450 nm was measured in Pherastar plate reader (BMG).

### Mass Spectrometry

NanoLC coupled to Electrospray Quadrupole Time-of-Flight (ESI-QTOF, Bruker, Karlsruhe, Germany) was applied to identify the biomaterial before and after ensilication.

### Thermogravimetric analysis

TGA on ensilicated lysozyme was carried out as follows: The sample, with a starting weight of 6.45 mg, was heated up to 800 °C at 10 °C/min under a 20 mL/min flow of nitrogen in a Thermogravimetry (Setaram Setsys Evolution 16 TGA-DTA-DSC) instrument.

### Statistical analysis

Data from the lysozyme activity assays were analysed using two tailed un-paired t-tests. p values < 0.05 were considered statistically significant. ELISA data were analysed with non-parametric test, Kruskal-Wallis (IBM SPSS 22). p values < 0.05 were considered significant.

## Additional Information

**How to cite this article:** Chen, Y.-C. *et al*. Thermal stability, storage and release of proteins with tailored fit in silica. *Sci. Rep.* **7**, 46568; doi: 10.1038/srep46568 (2017).

**Publisher's note:** Springer Nature remains neutral with regard to jurisdictional claims in published maps and institutional affiliations.

## Supplementary Material

Supplementary Information

## Figures and Tables

**Figure 1:**
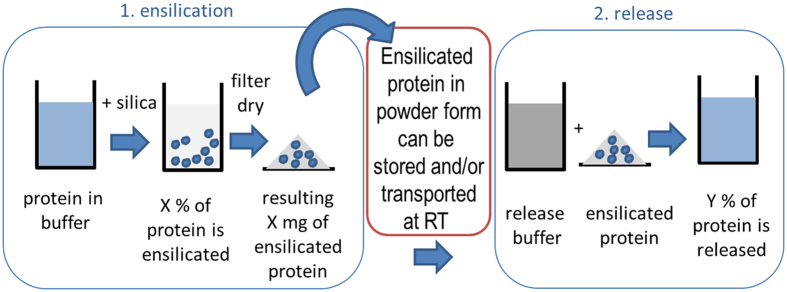
Ensilication and subsequent release methods schematic.

**Figure 2:**
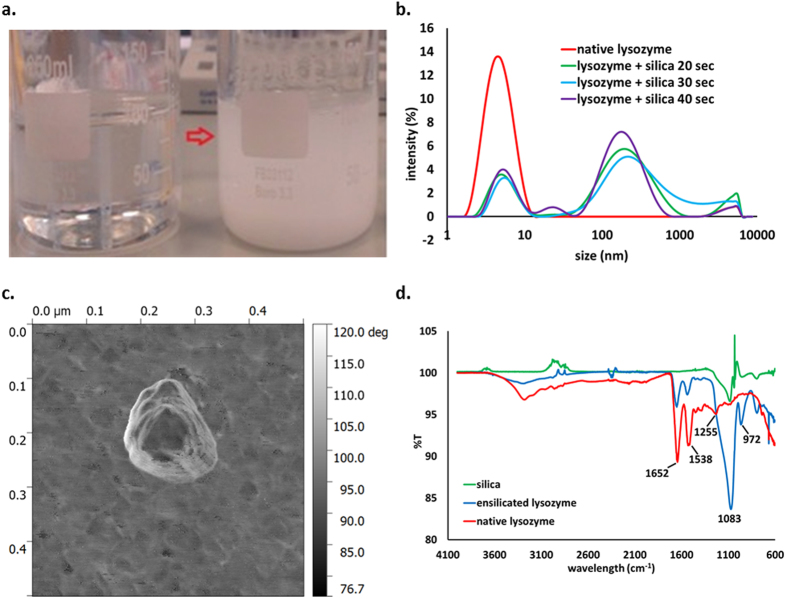
Formation of ensilicated powder. (**a**) Illustration of the ensilication. Left beaker contains protein solution, right beaker – protein solution with prehydrolysed TEOS after the formation of the silica precipitate. (**b**) DLS data for native lysozyme (red), and immediately after addition of TEOS (green – in 20 s, blue - in 30 s, purple – in 40 s). (**c**) AFM image of ensilicated lysozyme. (**d**) FT-IR spectra for native lysozyme (red), silica (green) and ensilicated lysozyme (blue). The bands at 1652, 1538, 1255 cm^−1^ correspond to amide I, amide II and amide III groups in lysozyme and the bands at 1063 and 972 cm^−1^ correspond to Si-O-Si stretching and Si-O bending.

**Figure 3:**
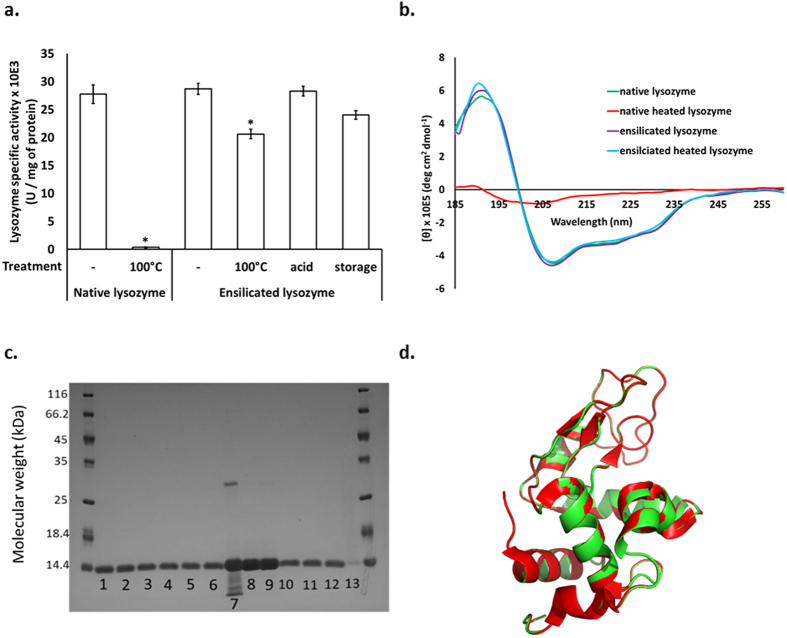
Characterisation of the ensilicated and released lysozyme. (**a**) Specific activity of lysozyme protein before and after ensilication. Ensilicated lysozyme powder was treated by heating at 100 °C for 5 h, by incubating in 10 M HCl for 3 h, or stored at 22 °C for 6 months. At the end of the treatment, the ensilicated lysozyme was released and the lysozyme activity measured. Data are mean values and SEM, n = 3, *p < 0.05. (**b**) CD spectra for lysozyme (green - native lysozyme; red - native heated lysozyme; purple – ensilicated lysozyme; blue - ensilicated heated lysozyme). (**c**) SDS-PAGE analysis of lysozyme. Lanes 1–3: ensilicated lysozyme (3 individual preparations), Lanes 4–6: ensilicated heated lysozyme (3 individual preparations), Lane 7: heated native lysozyme solution, Lane 8: native lysozyme solution (a control), Lane 9: dry-heated lyophilised lysozyme, Lanes 10–12: ensilicated acid treated lysozyme (3 individual preparations), Lane 13: supernatant after ensilication. (**d**) Ribbon diagram of the crystal structure of the released lysozyme (green) superimposed onto a published structure of the protein (red; PDB code 2w1x). Rmsd overall 0.09 Å. Rmsd individual amino acids 0.24 Å.

**Figure 4:**
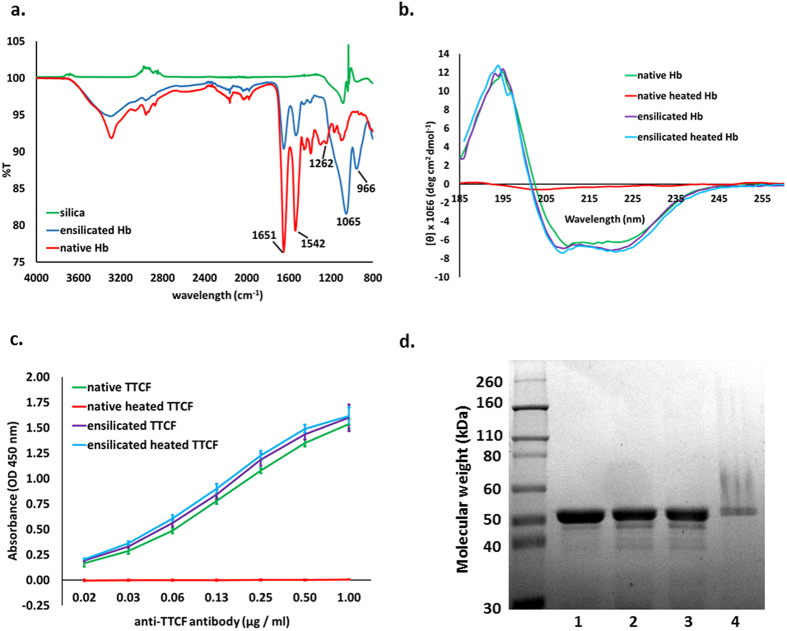
Structural data for ensilication of haemoglobin (Hb) and TTCF. (**a**) FT-IR data for Hb, silica, and ensilicated Hb (native Hb – red, silica – green and enslicated Hb – blue). The bands are as described in Fig. 2d. (**b**) Circular dichroism data on Hb samples: native Hb (green), native Hb after heat treatment (red), ensilicated Hb (purple), and Hb released from ensilication after heat treatment (blue). (**c**) TTCF antibody binding capacity before and after ensilication. ELISA binding assay was performed on native TTCF (green), native TTCF heated for 2 h at 80 °C (red), TTCF release from ensilication (purple) and ensilicated TTCF heated for 2 h at 80 °C and released (blue). Data are mean values and SEM, n = 3. Kruskal-Wallis non-parametric test comparison revealed no significant difference between the native TTCF and the two ensilicated and released samples (p = 0.834), but revealed a significant difference to the native heat-treated TTCF (p = 0.002). (**d**) SDS-PAGE analysis of TTCF. Lane 1 – native TTCF, Lane 2 – ensilicated and released TTCF, Lane 3 – ensilicated TTCF heated for 2 h at 80 °C and released, Lane 4 – TTCF heated in solution for 2 h at 80 °C. Representative images from 3 independent preparations.

## References

[b1] WHO. Immunization, Vaccines and Biologicals http://www.who.int/immunization/en/ (2014).

[b2] Kendal A. P., Snyder R. & Garrison P. J. Validation of cold chain procedures suitable for distribution of vaccines by public health programs in the USA. Vaccine 15, 1459–1465 (1997). 9302761 10.1016/s0264-410x(97)00060-1

[b3] Brandau D. T., Jones L. S., Wiethoff C. M., Rexroad J. & Middaugh C. R. Thermal stability of vaccines. Journal of Pharmaceutical Sciences 92, 218–231, doi: 10.1002/jps.10296 (2003). 12532371

[b4] WHO. Temperature sensitivity of vaccines http://www.who.int/vaccines-documents/DocsPDF06/847.pdf(2006).

[b5] Setia S. . Frequency and causes of vaccine wastage. Vaccine 20, 1148–1156, doi: 10.1016/S0264-410X(01)00433-9 (2002). 11803076

[b6] Alcock R. . Long-Term Thermostabilization of Live Poxviral and Adenoviral Vaccine Vectors at Supraphysiological Temperatures in Carbohydrate Glass. Sci. Transl. Med. 2, doi: 10.1126/scitranslmed.3000490 (2010). 20371486

[b7] Amorij J. P. . Towards tailored vaccine delivery: Needs, challenges and perspectives. J. Control. Release 161, 363–376, doi: 10.1016/j.jconrel.2011.12.039 (2012). 22245687

[b8] Arora V. Nanotechnology Drug Delivery Systems: An Insight http://trialx.com/curetalk/2012/10/nanotechnology-drug-delivery-systems-an-insight/(2013).

[b9] Prow T. W. . Nanopatch-Targeted Skin Vaccination against West Nile Virus and Chikungunya Virus in Mice. Small 6, 1776–1784, doi: 10.1002/smll.201000331 (2010). 20665754

[b10] Fu K., Klibanov A. M. & Langer R. Protein stability in controlled-release systems. Nat Biotechnol 18, 24–25, doi: 10.1038/71875 (2000). 10625383

[b11] Diwan M. & Park T. G. Pegylation enhances protein stability during encapsulation in PLGA microspheres. J Control Release 73, 233–244 (2001). 11516501 10.1016/s0168-3659(01)00292-9

[b12] Crotts G. & Park T. G. Stability and release of bovine serum albumin encapsulated within poly(D, L-lactide-co-glycolide) microparticles. J. Control. Release 44, 123–134, doi: Doi 10.1016/S0168-3659(96)01511-8 (1997).

[b13] Foo C. W. P., Huang J. & Kaplan D. L. Lessons from seashells: silica mineralization via protein templating. Trends in Biotechnology 22, 577–585, doi: 10.1016/j.tibtech.2004.09.011 (2004). 15491802

[b14] Perry C. C. Silicification: The processes by which organisms capture and mineralize silica. Rev Mineral Geochem 54, 291–327, doi: 10.2113/0540291 (2003).

[b15] Tacke R. Milestones in the biochemistry of silicon: From basic research to biotechnological applications. Angew Chem Int Edit 38, 3015–3018, doi: 10.1002/(Sici)1521-3773(19991018)38:20<3015::Aid-Anie3015>3.3.Co;2-X (1999). 10540406

[b16] Delalat B. . Targeted drug delivery using genetically engineered diatom biosilica. Nat Commun 6, doi: 10.1038/ncomms9791 (2015). 26556723

[b17] Hansen D. E. Recent developments in the molecular imprinting of proteins. Biomaterials 28, 4178–4191, doi: 10.1016/j.biomaterials.2007.06.017 (2007). 17624423

[b18] Henry E. R. . Experiments on Hemoglobin in Single Crystals and Silica Gels Distinguish among Allosteric Models. Biophysical Journal 109, 1264–1272, doi: 10.1016/j.bpj.2015.04.037 (2015). 26038112 PMC4576146

[b19] Urabe Y. . Encapsulation of hemoglobin in mesoporous silica (FSM)-enhanced thermal stability and resistance to denaturants. Chembiochem 8, 668–674, doi: 10.1002/cbic.200600486 (2007). 17330900

[b20] Eggers D. K. & Valentine J. S. Crowding and hydration effects on protein conformation: a study with sol-gel encapsulated proteins. J Mol Biol 314, 911–922, doi: 10.1006/jmbi.2001.5166 (2001). 11734007

[b21] Eggers D. K. & Valentine J. S. Molecular confinement influences protein structure and enhances thermal protein stability. Protein Sci 10, 250–261, doi: 10.1110/ps.36201 (2001). 11266611 PMC2373941

[b22] Makoff A. J., Oxer M. D., Romanos M. A., Fairweather N. F. & Ballantine S. Expression of tetanus toxin fragment C in E. coli: high level expression by removing rare codons. Nucleic Acids Research 17, 10191–10202 (1989). 2690015 10.1093/nar/17.24.10191 PMC335293

[b23] Mitra A. & Rimstidt J. D. Solubility and dissolution rate of silica in acid fluoride solutions. Geochim Cosmochim Ac 73, 7045–7059, doi: 10.1016/j.gca.2009.08.027 (2009).

[b24] Barth A. Infrared spectroscopy of proteins. Biochimica et Biophysica Acta (BBA) - Bioenergetics 1767, 1073–1101, doi: 10.1016/j.bbabio.2007.06.004 (2007). 17692815

[b25] Saadoun M. . Formation of luminescent (NH4)2SiF6 phase from vapour etching-based porous silicon. Applied Surface Science 210, 240–248, doi: 10.1016/S0169-4332(03)00152-1 (2003).

[b26] Mathe C. . Structural determinants for protein adsorption/non-adsorption to silica surface. PLoS One 8, e81346, doi: 10.1371/journal.pone.0081346 (2013). 24282583 PMC3839912

[b27] Gessner B. D., Beller M., Middaugh J. P. & Whitford G. M. Acute fluoride poisoning from a public water system. The New England Journal of Medicine 330, 95–99, doi: 10.1056/NEJM199401133300203 (1994). 8259189

[b28] Hewitt E. W. . Natural processing sites for human cathepsin E and cathepsin D in tetanus toxin: implications for T cell epitope generation. Journal of Immunology 159, 4693–4699 (1997). 9366392

[b29] Elkordy A. A., Forbes R. T. & Barry B. W. Integrity of crystalline lysozyme exceeds that of a spray-dried form. International Journal of Pharmaceutics 247, 79–90, doi: 10.1016/S0378-5173(02)00379-4 (2002). 12429487

[b30] Forsythe E. L. & Pusey M. L. The effects of acetate buffer concentration on lysozyme solubility. Journal of Crystal Growth 168, 112–117, doi: 10.1016/0022-0248(96)00368-5 (1996).

[b31] Yoshioka S., Aso Y., Izutsu K. & Terao T. The effect of salts on the stability of beta-galactosidase in aqueous solution, as related to the water mobility. Pharmaceutical Research 10, 1484–1487 (1993). 8272411 10.1023/a:1018931527176

[b32] Antoniou A. N. & Watts C. Antibody modulation of antigen presentation: positive and negative effects on presentation of the tetanus toxin antigen via the murine B cell isoform of FcgammaRII. European Journal of Immunology 32, 530–540, doi: 10.1002/1521-4141(200202)32:2<530::AID-IMMU530>3.0.CO;2-X (2002). 11828370

