# Supplementary material for: Thermal stability, storage and release of proteins with tailored fit in silica
Source: Sci Rep. 2017 Apr 24;7:46568. doi: 10.1038/srep46568 (PMC5402271; doi:10.1038/srep46568)
Supplement: Supplementary Information [file srep46568-s1.pdf]

**Supplementary information for:**

# Thermal stability, storage and release of proteins with tailored fit in silica

Yun-Chu Chen[1], Tristan Smith[2], Robert Hicks[2], Aswin Doekhie[1], Francoise Koumanov[3], Stephen A. Wells[4], Karen J. Edler[1], Jean van den Elsen[3], Geoffrey D. Holman[3], Kevin J. Marchbank[5], Asel Sartbaeva[1*].

[1] Department of Chemistry; [2] Centre for Sustainable Chemical Technologies, Department of Biology and Biochemistry, [3] Department of Biology and Biochemistry; [4] Department of Chemical Engineering, University of Bath, Bath, BA2 7AY, UK; [5]Institute of Cellular Medicine, Newcastle University, The Medical School, Framlington Place, Newcastle upon Tyne, NE2 4HH, UK

All correspondence and requests should be addressed to A.S. (a.sartbaeva@bath.ac.uk).

## Supplementary Methods

### Crystallisation of released lysozyme

Crystallisation of lysozyme released from silica was achieved with use of the 'hanging drop' vapour diffusion technique. Pure lysozyme has been crystallised on many occasions before and the conditions mentioned here have been adapted from literature for use within this procedure. Released lysozyme was dialysed in 0.1 M sodium acetate pH 4.6 and concentrated to 25 mg/ml. In a 24-wells crystallisation plate, 700 µl of 1.5 M NaCl in 0.1 M sodium acetate pH 4.6 was added to each reservoir. The lysozyme solution was mixed 1:1 on a siliconized coverslip with reservoir solution creating a 2 µl droplet. Diffusion within the covered well provided changes in the precipitant causing the reservoir solution to retain more water, thus providing the formation of crystals within the droplet as the protein concentration increased, until equilibrium was obtained. Crystals were formed after approximately 5 days incubation at 18°C.

### Choice of temperatures for thermal studies

Thermal parameters were based upon published Tm or observed inactivation temperatures. Lysozyme has a Tm of 74.8 ± 0.4 °C (1) in water, Hb (horse sigma) 72 °C (2) and TTCF shows rapid decrease in activity at 60°C (3). Ensilicated lysozyme and Hb were incubated at Tm+20 °C = approx. 100 °C and TTCF at Tm+20°C = 80 °C. Temperatures of 20 °C above the denaturing threshold assures proper stress testing of the ensilicated material. Visual inspection of unprotected protein in solution showed precipitation after thermal exposure.


1. Knubovets T, Osterhout JJ, Connolly PJ, Klibanov AM. Structure, thermostability, and conformational flexibility of hen egg-white lysozyme dissolved in glycerol. Proceedings of the National Academy of Sciences of the United States of America. 1999;96(4):1262-1267.
2. Suh, Y., Kim, B.J., Tam, K.C. et al. J Therm Anal Calorim (2014) 115: 2159. doi:10.1007/s10973-013-3424-5
3. Cohen H, van Ramshorst JD, Tasman A. Consistency in potency assay of tetanus toxoid in mice. Bulletin of the World Health Organization, 1959, 20: 1133-1150.

## Supplementary Figures

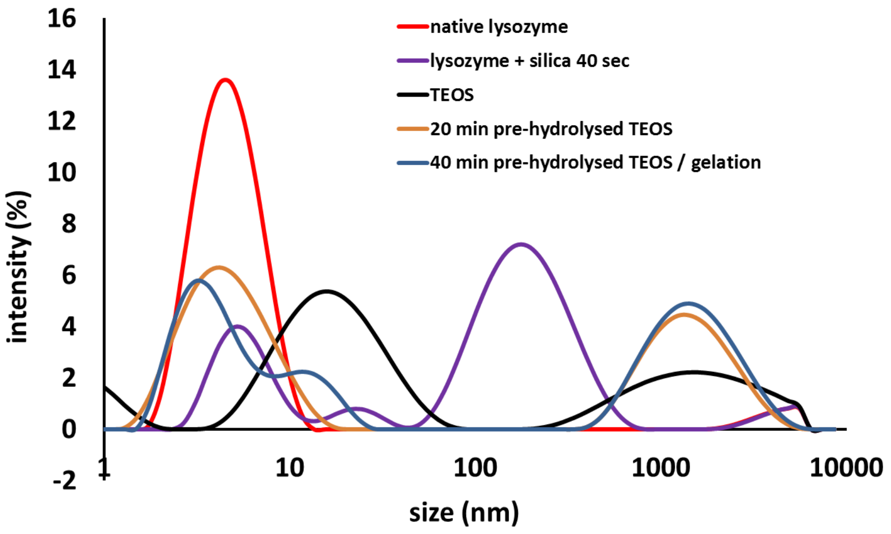


**Supplementary Figure S1: DLS data for TEOS (black), and prehydrolysed TEOS for 20 min (yellow) and 40 min (blue) reaching gelation state.** Data for native lysozyme (red) and Lysozyme + silica 40 sec (purple) were taken from Fig. 2b

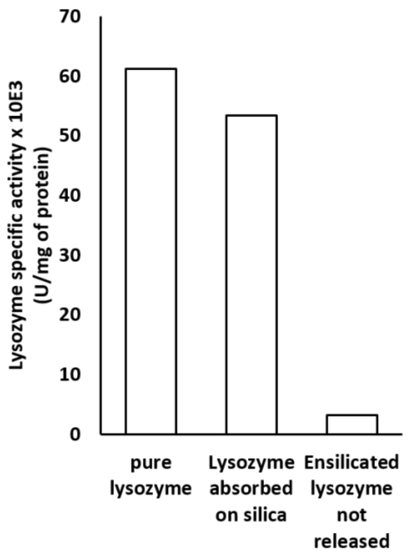


**Supplementary Figure S2:** Lysozyme activity assay of lysozyme absorbed onto silica or ensilicated lysozyme that has not been released. Native lysozyme solution (1mg/ml) was used as a control. Additional numerical data are provided in Supplementary Table S3.

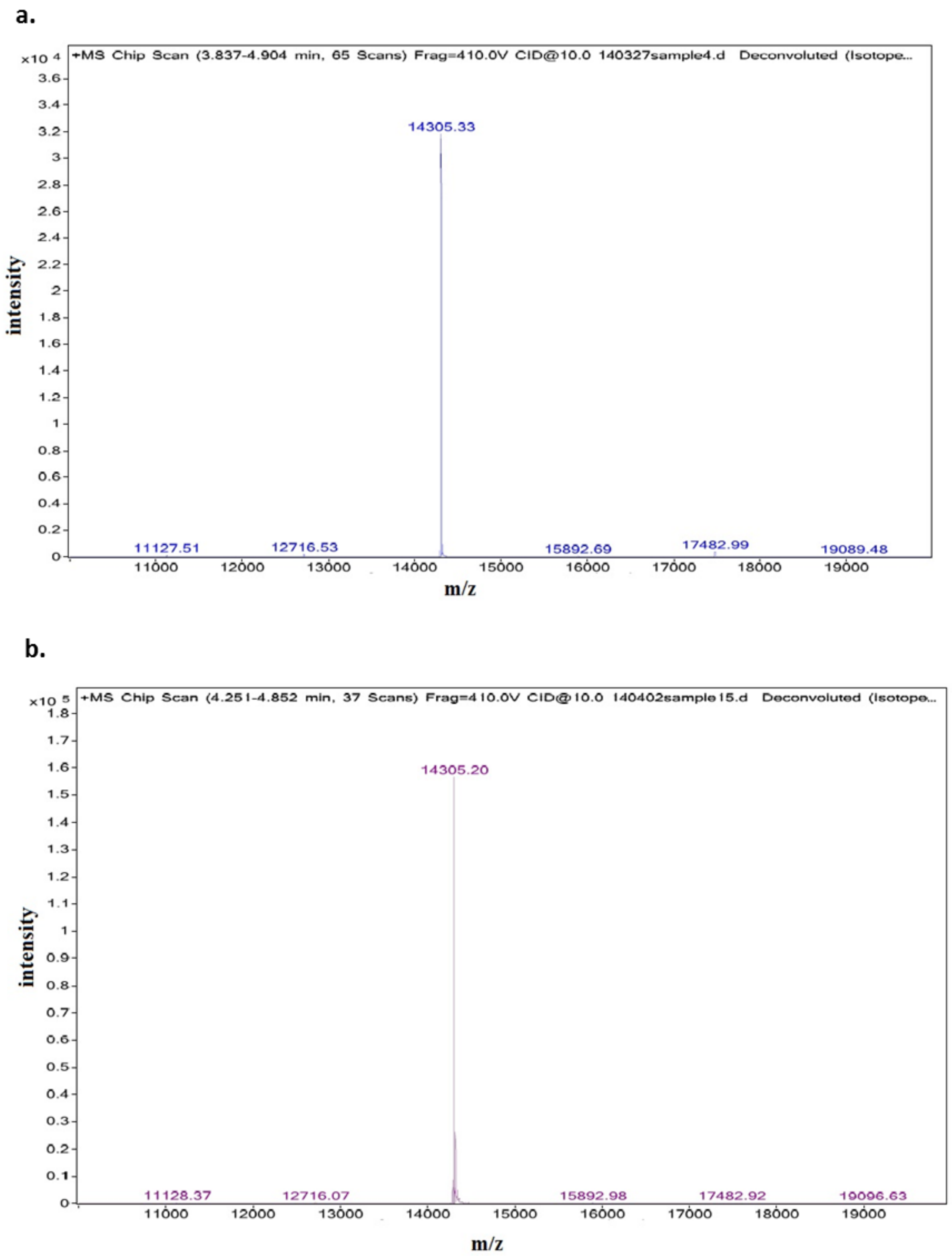


**Supplementary Figure S3:** Mass spectrometry analysis of lysozyme before (a) and after ensilication (b).

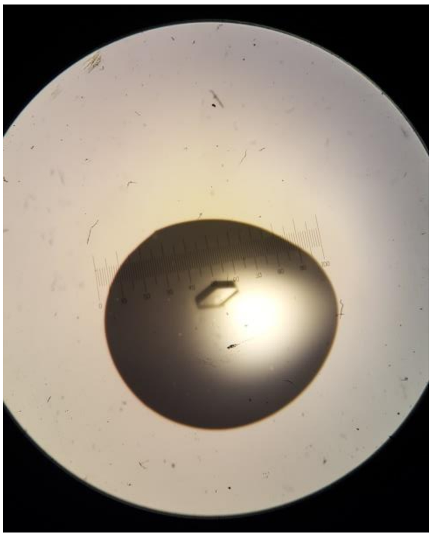

**Supplementary Figure S4:** Lysozyme crystallised after ensilication and release. Scale bar 100 µm.

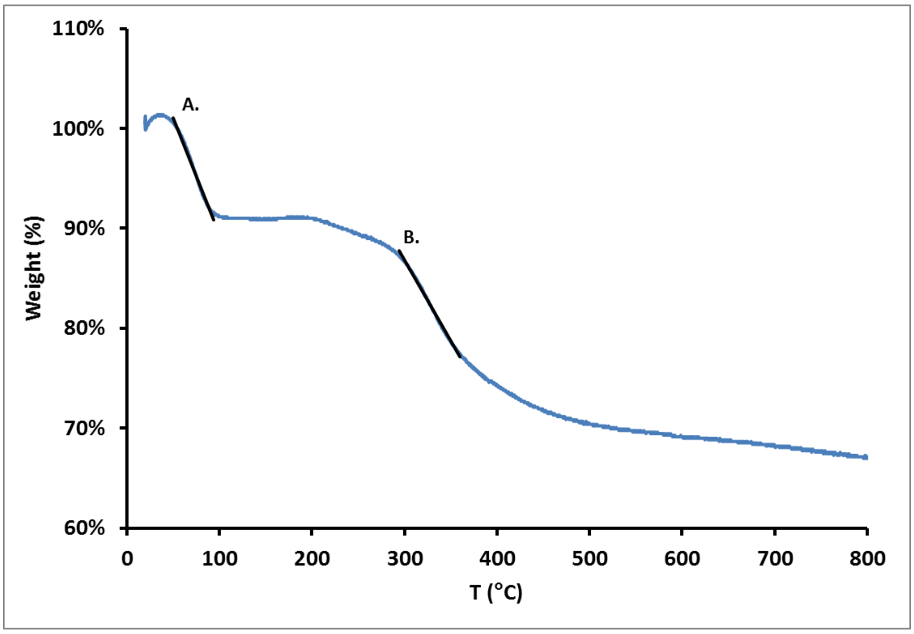


**Supplementary Figure S5: Thermo-Gravimetric Analysis (TGA) analysis of the decomposition of ensilicated lysozyme with an inert sample purge gas.** The sample, with a starting weight of 6.45 mg, was heated up to 800 °C at 10 °C /min under a 20 mL/min flow of nitrogen in a Thermogravimetry (Setaram Setsys Evolution 16 TGA-DTA-DSC) instrument. The water weight loss of 0.57 +/-1 mg started at 50 °C and continued evolving until 100 °C (line A), a weight loss of 8.9 +/- 0.1%. The result is consistent with a previous study reporting that lysozyme starts to decompose at 273 °C (line B). (Elkordy, A. A., Forbes, R. T. and Barry, B. W., 2002. Integrity of crystalline lysozyme exceeds that of a spray- dried form. International Journal of Pharmaceutics, 247, 79-90.)

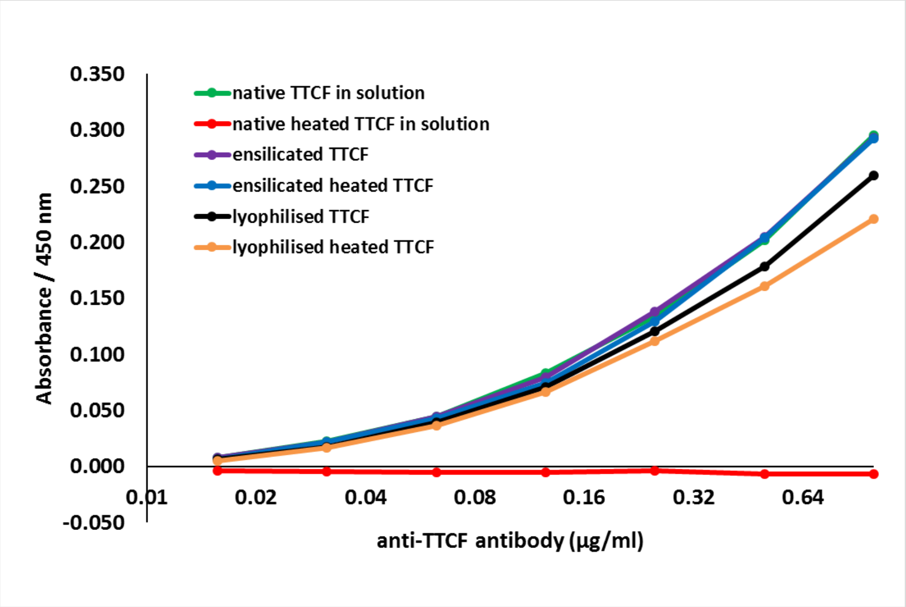


**Supplementary Figure S6: Comparison of antibody binding capacity of lyophilised recombinant TTCF and recombinant TTCF in solution.** ELISA binding assay was performed on native TTCF (green), native TTCF heated for 2 h at 80°C (red), TTCF release from ensilication (purple) and ensilicated TTCF heated for 2h at 80°C and released (blue), lyophilised TTCF(black) and lyophilised TTCF heated for 2 h at 80°C (yellow).

**Supplementary Tables:**

**Table S1**

**Efficiency of ensilication for lysozyme.** The lysozyme ensilication efficiency was determined using three independently prepared samples (A, B and C). To do this, approximately 100 mg of lysozyme was ensilicated, yielding on average 170.9 mg of ensilicated lysozyme powder. Following this, a known quantity of each sample (approximately 5 mg) was released and the protein concentration was determined with BCA protein assay kit and overall efficiency was then calculated by extrapolating the amount of protein released in this sample, to the total amount of ensilicated powder yielded from the initial preparation. Efficiency was determined to be 93 ± 2.3%.

| | Sample A | Sample B | Sample C | Average | Standard Deviation |
|---|---|---|---|---|---|
| Lysozyme used for ensilication (mg) | 100.2 | 100.1 | 100.2 | 100.2 | 0.1 |
| Ensilicated powder yielded (mg) | 172.4 | 171.0 | 169.3 | 170.9 | 1.6 |
| Ensilicated powder used for release (mg) | 5.1 | 5.3 | 5.1 | 5.2 | 0.1 |
| Protein concentration released (mg/mL) | 2.8 | 2.8 | 2.8 | 2.8 | 0.0 |
| Total lysozyme ensilicated (mg) | 95.5 | 90.8 | 93.4 | 93.2 | 2.3 |
| Ensilication efficiency (%) | 95.3% | 90.7% | 93.2% | 93.0% | 2.3% |
| Total efficiency = 93.0% ± 2.3% | | | | | |

**Table S2**

**Control experiments for lysozyme activity of native and ensilicated material.** All activities were measured using the EnzChek® Lysozyme Assay Kit and the protein concentrations with BSA protein assay kit and confirmed by UV 280 nm absorption reading in UV spectrophotometer. Example data with coefficients of variation estimated based on 3-4 technical replications.

| Conditions | Activity (U / ml) | CV (%) | Protein concentration (mg / ml) | Specific activity (U / mg) |
|---|---|---|---|---|
| Native lysozyme | 60655 | 10.2 | 1.300 | 46658 |
| Native lysozyme incubated in 10 M HCl for 3 h | N.D. | - | 0.130 | N.D. |
| Ensilicated lysozyme in Tris buffer* | 507 | 4.5 | N.D. | N.D. |
| Ensilicated released in 190 mM NaF (pH 9) | N.D. | - | N.D. | N.D. |
| Ensilicated released in 0.1 M HCl (pH 1.0) | N.D. | - | 0.065 | N.D. |
| Ensilicated released in 190 mM NaF, pH with HCl to pH 6.0 | N.D. | - | N.D. | N.D. |
| Ensilicated released in 190 mM NaF, pH with HCl to pH 5.0 | 4077 | 10.5 | 0.084 | 48540 |
| Ensilicated released in 190 mM NaF, pH with HCl to pH 4.0 | 10739 | 2.1 | 0.271 | 39629 |
| Ensilicated released in 190 mM NaF, pH with HCl to pH 3.0 | 7844 | 8.4 | 0.263 | 29826 |
| Ensilicated released in 190 mM NaF, pH with HCl to pH 2.0 | 8433 | 4.18 | 0.296 | 28491 |

CV (%) – coefficient of variation (percent); N.D. – no detectable activity or concentration.

*We attribute this small activity to residual lysozyme adhering to the silica surface.

**Table S3**

**X-ray data collection statistics**

| Data collection | | | |
|---|---|---|---|
| Space Group | P4322 | | |
| Unit cell dimensions (a,b,c Å) | 78.60 | 78.60 | 37.04 |
| (α=β=γ ˚) | 90.00 | | |
| Mosaicity | 0.72 | | |
| | | | |
| Resolution range (Å; last shell) | 55.58 - 1.75 | | (1.82 - 1.75) |
| Total number of reflections | 101347 | | |
| Number of unique reflections | 11498 | | |
| Average redundancy (last shell) | 8.81 | | (5.96) |
| Completeness (%; last shell) | 94.7 | | (88.6) |
| R-merge (last shell) | 0.039 | | (0.128) |
| R-meas (last shell) | 0.041 | | (0.140) |
| R-measA (I+, I- reflns kept apart) (last shell) | 0.04 | | (0.144) |
| Reduced Chi-Squared (last shell) | 0.95 | | (0.69) |
| Output <I/sigI> (last shell) | 37.6 | | (9.1) |

**Table S4**

**Model refinement statistics**

| Refinement | |
|---|---|
| Resolution range (Å) | 30.822 - 1.754 |
| Number of protein atoms | 1054 |
| Number of solvent atoms | 150 |
| $R_{work}$ (%) | 0.149 |
| $R_{free}$ (%) | 0.1944 |
| RMS deviations | |
| Bonds (Å) | 0.0089 |
| Angles (°) | 1.181 |
| Molprobity | 1.22 |
| Ramachandran | |
| Favoured regions (%) | 98.47 |
| Allowed (%) | 1.53 |
| Outliers (%) | 0.00 |
